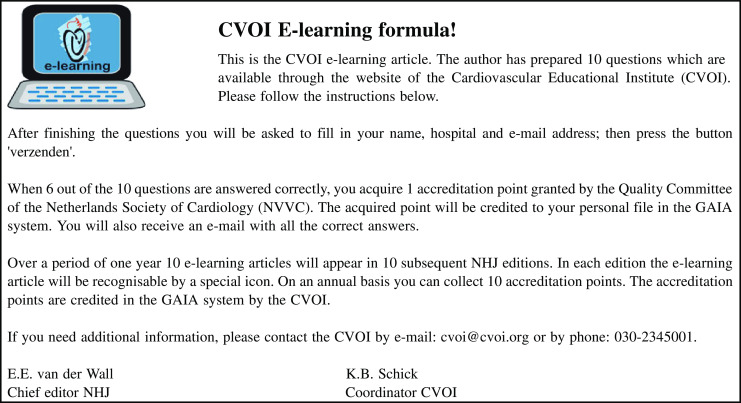# Infective complications after transcatheter aortic valve implantation: results from a single centre

**DOI:** 10.1007/s12471-012-0303-9

**Published:** 2012-08-14

**Authors:** K. Onsea, P. Agostoni, M. Voskuil, M. Samim, P. R. Stella

**Affiliations:** Division of Cardiology, University Medical Centre Utrecht, UMC Utrecht, Heidelberglaan 100, Utrecht, Postbus 85500, 3508 GA Utrecht, the Netherlands

**Keywords:** TAVI, Complication, Infection

## Abstract

After its first introduction in 2002, transcatheter aortic valve implantation (TAVI) has continuously gained more foothold for the treatment of severe aortic stenosis and is nowadays a viable treatment option for inoperable patients or patients at high risk for conventional surgical aortic valve replacement. Although ideally carried out in a so-called hybrid room, incorporating both the strict hygiene and advanced life support possibilities of the operating theatre and the imaging and percutaneous arsenal of the catheterisation suite, in most centres TAVI is at present performed in the catheterisation laboratory. This may raise concern about an increased risk of infection, since there the criteria that are applied regarding disinfection and sterilisation are not as stringent as those of the operating theatre. Therefore, we retrospectively assessed the number of infective complications in patients undergoing TAVI in the catheterisation lab of our institution. Eleven out of 73 patients developed a postprocedural infection, one of which could be attributed to the procedure itself, being superinfection of a surgical groin cut-down. Our conclusion is that percutaneous aortic valve implantation in a catheterisation laboratory is not associated with an increased risk of infective complications.

## Introduction

The treatment of aortic stenosis has been revolutionised by the introduction of percutaneously insertable valve prostheses, which have been demonstrated to be a viable treatment option for inoperable patients or patients at high risk for conventional surgical valve replacement [1, 2]. Two CE approved transcatheter bio-prosthetic aortic valves (the Edwards Sapien balloon expandable valve (Edwards Lifescience, Inc., Irvine, CA, USA) and the Medtronic self-expandable CoreValve (Medtronic CV, Luxembourg)) are currently on the market, which can be implanted via a transfemoral (suitable for both types of valves), transapical (Edwards valve only), subclavian (Medtronic CoreValve only) or, as recently reported, direct transaortic approach [3] during a transcatheter aortic valve implantation (TAVI). The decision whether a patient is treated with a percutaneous or surgical valve should be discussed and made in the heart team [4], a multidisciplinary meeting where invasive and non-invasive cardiologists, cardiac surgeons and ideally representatives from other disciplines involved such as an anaesthesiologist and/or geriatrician, discuss the optimal strategy to come to a patient-tailored therapy. At present, it is generally accepted that surgical valve replacement still represents the gold standard for degenerated aortic valve stenosis, whereas TAVI should be restricted to those patients too ill to be operated on. Nevertheless, expectations are that in the foreseeable future, if early benefit of TAVI is confirmed during long-term follow-up, percutaneous valve treatment will become available for a progressively broader patient population, including low-risk surgical patients. This shift from surgical to transcatheter valve implantation not only entails a shift from the cardiac surgeon to the interventional cardiologist as the primary operator, but also frequently implies a move from the operating theatre to the catheterisation room. There may be concerns that a non-surgical environment might imply less stringent hygienic and sterile precautions, thereby increasing the risk for procedure-related and prosthesis infections, especially in this highly vulnerable patient group. Remmelts et al. already demonstrated that the incidence of infection of an implantable cardioverter-defibrillator did not differ between implantation in the operating room and implantation in the cardiac catheterisation laboratory [5]. In a similar manner, our aim was to retrospectively evaluate the rate of infective complications of any kind in patients undergoing percutaneous aortic valve replacement in the catheterisation suite of our institution.

## Methods

### Data collection

Electronic patient records from patients undergoing transfemoral or transapical TAVI from August 2008 to July 2011 were systematically reviewed. The following data were collected: patient characteristics, comorbidities and cardiac history; procedural characteristics, including access site, type of valve (brand and size) and performance of surgical groin cut-down; occurrence of postoperative complications for up to 30 days and 30-day survival. Complications were classified into eight different categories. For the aim of this study, we specifically looked at infections, which were further classified according to site of origin (as noted in the electronic patient record). Whenever possible, further information about causal pathogen and treatment was assembled.

### Aortic valve implantation

TAVI procedures were routinely performed by a team, consisting of two interventional cardiologists, one cardiologist specialised in echocardiography, one cardiac surgeon, one anaesthetist and two specialised nurses. The procedure took place in the catheterisation suite, which was cleaned the evening before the TAVI procedure and only accessible for persons wearing appropriate scrubs, masks, caps and, if standing at the operating table, gowns. At the beginning of the procedure, meticulous hand washing and donning of sterile gloves was done by each member of the operating team, and the patient was prepped and draped in a sterile fashion. Forty-five patients were treated via a transfemoral route, the remaining 28 underwent a transapical approach according to standard techniques as described earlier [6, 7]. In the early cohort of 27 patients, the right or left common femoral artery and vein were surgically exposed at the beginning of the procedure in case emergency cardiopulmonary bypass was needed.

With growing operator experience, this practice was abandoned and a complete percutaneous access became the gold standard in the subsequent 46 patients. All patients were pre-treated with aspirin and clopidogrel and received heparin during the procedure in order to maintain an activated clotting time above 250 ms. All patients received antibiotic prophylaxis (first- or third-generation cephalosporin or vancomycin). The size of the valve was chosen according to echocardiographic estimate of aortic annulus diameter and varied between 23 (24 patients), 26 (44 patients) and 29 mm (5 patients). All procedures were done under general anaesthesia and with the aid of fluoroscopic and transoesophageal echocardiographic guidance for accurate valve deployment.

The stenotic valve was predilated with an undersized balloon to facilitate valve implantation and subsequent valve placement was accomplished during rapid pacing in the right ventricle or epicardium. If necessary, additional post-dilation was performed in case of relevant para-valvular regurgitation.

## Results

### Patients and procedures (Tables 1, 2)

**Table 1:** Patient characteristics and cardiac history

Clinical characteristics	N (=73)
Male sex	28 (38.3 %)
Age (years)	74.97 (±7.2)
Log EuroSCORE (%)	18 (±10.41)
Diabetes	22 (30.1 %)
COPD	14 (19.1 %)
Severe renal insufficiency^a^	14 (19.1 %)
Active malignancy	5 (6.8 %)
Peripheral vascular disease	20 (27.4 %)
Porcelain aorta	10 (13.7 %)
Previous stroke	13 (17.8 %)
Cardiac history	
Previous myocardial infarction	20 (27.4 %)
Previous PCI	22 (30.1 %)
Previous CABG	15 (20.5 %)
Previous cardiac surgery other than CABG	3^b^ (4.1 %)
Left ventricular ejection fraction <35 %	7 (9.6 %)
Moderate/severe mitral insufficiency^c^	14 (19.2 %)
Pulmonary hypertension^d^	26 (35.6 %)

*CABG* coronary artery bypass grafting; *COPD* chronic obstructive pulmonary disease, *PCI* percutaneous coronary intervention

All data are presented as mean ± standard deviation or number (percentage)

^a^Estimated glomerular filtration rate <30 ml/min/1.73 m^2^

^b^2 pericardiectomies - 1 mitral valve plasty

^c^Echocardiographic estimate of ≥2/4 mitral regurgitation

^d^Echocardiographic measurement of pulmonary artery systolic pressure >35 mmHg

**Table 2:** Procedural details and 1-month survival rate

Procedural characteristics	
TF/TA	45/28 (61.6/38.4 %)
Edwards/CoreValve	67/6 (91.7/8.2 %)
Valve size 23/26/29	24/44/5 (32.9/60.3/6.8 %)
Surgical groin exposition	27 (37 %)
Outcome	
1-month survival	62 (84.93 %)

*TF/TA* transapical/transfemoral

All data are presented as number (percentage)

In a time span of almost 3 years, 73, predominantly female, patients, received a percutaneous aortic valve. Mean age and logistic EuroSCOREs were 75 (±7.2) and 18 (±10.41) respectively. Comorbidities included diabetes (30.1 %), chronic obstructive pulmonary disease (19.1 %), renal insufficiency (19.1 %), previous stroke (17.8 %), peripheral vascular disease (27.4 %), porcelain aorta (13.7 %) and active malignancy (6.8 %). Seven patients (9.6 %) had a severely depressed left ventricular systolic function; significant mitral insufficiency was present in 14 patients (19.2 %) and 26 (35.6 %) suffered from pulmonary hypertension. Forty-five patients (61.6 %) were treated via the transfemoral approach, the remaining 28 (38.4 %) via trans-apical access. One-month survival approached 85 %.

### Procedural and infective complications (Tables 3, 4, 5)

**Table 3:** Incidence of periprocedural complications

Procedural complications	
Vascular	15 (20.6 %)
Valve dislocation	2 (2.7 %)
TIA or stroke	4 (5.5 %)
Infective	11(15.1 %)
Rhythm	13 (17.8 %)
Tamponade	1 (1.4 %)
Contrast nephropathy	2 (2.7 %)
Non-access site bleeding	1 (1.3 %)

All data are presented as number (percentage). *TIA* transient ischaemic attack

**Table 4:** Incidence of infective complications

Infective complications	N
Groin	1 (1.4 %)
UTI	5 (6.8 %)
Bronchopulmonary	3 (4.1 %)
Unknown	2 (2.7 %)

All data are presented as number (percentage). *UTI* urinary tract infection

**Table 5:** Characteristics of patients with infective complications

Patient	Infection	Approach	Log EuroSCORE	30-day survival
1	UTI	Transapical	1.8	Yes
2	UTI	Transfemoral	12.08	Yes
3	Unknown origin	Transfemoral	13.44	Yes
4	UTI	Transapical	29.02	Yes
5	Pneumonia	Transfemoral	36.78	No
6	UTI	Transapical	15.6	No
7	Pneumonia	Transapical	11.66	Yes
8	UTI	Transfemoral	11	Yes
9	Bronchitis	Transapical	21.95	Yes
10	Unknown origin	Transfemoral	10.88	Yes
11	Groin	Transapical	16	Yes

*UTI* urinary tract infection

Total number of complications was 49, which were classified into eight different categories. Vascular complications (including groin haematoma requiring transfusion, pseudo-aneurysm, aorto/ileo/femoral dissection or rupture) occurred in 15 patients (20.6 %); in 2 patients (2.7 %), the deployed valve dislocated and migrated, necessitating emergent surgery; incidence of transient ischaemic attack and stroke was 5.5 % (4 patients); tamponade (probably caused by perforation of the right ventricle by the temporary pacing wire) and major non-access site bleeding (haemothorax) each occurred in one patient; 2 patients suffered from contrast nephropathy (none requiring dialysis); 13 patients (17.8 %) had postprocedural rhythm disturbances (atrial fibrillation, severe bradycardia or high-grade atrioventricular block compelling pacemaker placement).

Eleven cases of infection were diagnosed during hospital stay, including 5 of the urinary tract, 3 bronchopulmonary infections, 1 in the groin and 2 infections of unknown cause. Microbiology data were reviewed, but a pathogen could only be isolated and identified (*Acinetobacter*) in the patient with the infected groin. All patients were (empirically) treated with broad-spectrum antibiotics; 9 patients had a good outcome, while 2 patients died. The first patient suffered from overwhelming sepsis due to bilateral pneumonia (confirmed at obduction) and died 2 days after TAVI, while the second patient, who had been treated for a urinary tract infection, suddenly died 23 days post-TAVI. No autopsy was performed in the last case.

## Discussion

Since the emergence of percutaneous valves, high-risk patients with degenerated aortic valve stenosis can be offered a less invasive alternative instead of conventional surgical valve replacement. Via the femoral, apical, subclavian or direct aortic approach, a transcatheter aortic bioprosthesis can be introduced, positioned and deployed inside the native valve, which itself is oppressed against the aortic wall. After its introduction in 2002 [8], the number of TAVI procedures has grown exponentially and its number will continue to increase, as more and more evidence becomes available supporting the benefit of a percutaneous approach in high-risk patients, not only compared with medical therapy, which entails a dismal prognosis in symptomatic aortic stenosis, but even when compared with the gold standard of surgery. Indications and suitability for TAVI should be formally discussed in the heart team, where surgery is balanced against this new transcatheter technique, for which long-term results regarding valve patency and patient survival are still lacking. Numerous factors (e.g. patient comorbidities and ‘frailty’, preferred access site, aortic annulus dimensions and calcification) should be taken into account to reach an optimal, individualised treatment plan.

In most centres, the insertion of a percutaneous aortic valve is carried out jointly by the cardiologist and cardiac surgeon, the first being traditionally skilled in catheter manipulations, the latter in creating access for apical or direct aortic approach. Ideally, TAVI procedures should be performed in a ‘hybrid’ room, an integration of cath lab and operating theatre where the traditional diagnostic functions of the cath lab are combined with the surgical functions of an operating room. However, at present, many hospitals that have started doing TAVI procedures do not have a hybrid lab, and therefore, TAVI procedures are often performed in the catheterisation lab.

Although strict sterile working conditions are pursued, high-efficiency particulate air filtered laminar airflow is absent in most cath labs and criteria regarding air control, room facilities and specific staff education are not as stringent as those of the operating theatre. Since this may increase the risk for procedure-related infection, we retrospectively evaluated the number of infections in 73 patients who underwent TAVI in our centre during a time span of almost three years.

We found 11 cases of infection, only one of which was directly related to the TAVI procedure, being superinfection of a surgically cut-down groin. This patient stemmed from the early patient cohort, during which the groin was ‘per protocol’ surgically exposed, in the event that emergent cardiopulmonary bypass was needed. Parallel with growing experience, this practice has been abandoned for an exclusive percutaneous approach. The 10 other cases of infections that were seen postprocedurally were of pulmonary, urinary or unknown origin, which can be anticipated in a high-risk patient population undergoing invasive procedures under general anaesthesia, whether these are performed in a catheterisation suite or operating theatre. Patients were treated with broad-spectrum antibiotics and had a favourable 1-month outcome, except for one patient who died of overwhelming sepsis due to bilateral pneumonia and another who suddenly succumbed 23 days postprocedure due to an unknown cause. It is reassuring that no cases of early prosthetic infection occurred in our patients. In the PARTNER (cohort B) trial (2), which is the largest randomised trial comparing TAVI and surgical valve replacement in high-risk patients, the location where TAVI was performed was not mentioned, nor was periprocedural infection specified as an endpoint. However, the incidence of endocarditis is reported, with a low incidence of prosthesis infection at 30 days (0 in TAVI patients and one in the surgical cohort) and one year (2 in the TAVI group, compared with 3 surgically implanted valve infections). Recently, we started to perform transfemoral TAVI using conscious sedation (thus avoiding intubation) and utilising intravascular instead of transoesophageal ultrasound guidance, hoping that this practice will result in improved and quicker patient recovery and thus less infection.

### Conclusion

In this single-centre registry the incidence of periprocedural infections complicating TAVI was low and only one case was directly related to the procedure itself, being superinfection of a surgically exposed groin. Although our study was observational and underpowered to detect the potential impact of cath lab versus operating theatre on infectious complications, it hints that percutaneous aortic valve implantation can be performed in a catheterisation laboratory without apparent increased risk of infective complications. Larger studies are needed to confirm this.